# Computed Tomography Angiography Identified High-Risk Coronary Plaques: From Diagnosis to Prognosis and Future Management

**DOI:** 10.3390/diagnostics14151671

**Published:** 2024-08-01

**Authors:** Kyriakos Dimitriadis, Nikolaos Pyrpyris, Panagiotis Theofilis, Emmanouil Mantzouranis, Eirini Beneki, Panagiotis Kostakis, George Koutsopoulos, Konstantinos Aznaouridis, Konstantina Aggeli, Konstantinos Tsioufis

**Affiliations:** First Department of Cardiology, School of Medicine, Hippokration General Hospital, National and Kapodistrian University of Athens, 11527 Athens, Greece; npyrpyris@gmail.com (N.P.); panos.theofilis@hotmail.com (P.T.); mantzoup@gmail.com (E.M.); e.beneki@hotmail.com (E.B.); panos_kost@hotmail.com (P.K.); giorgoskoutsopoulos93@gmail.com (G.K.); conazna@yahoo.com (K.A.); dina.aggeli@gmail.com (K.A.); ktsioufis@gmail.com (K.T.)

**Keywords:** coronary artery disease, atherosclerosis, vulnerable plaque, computed tomography, angiography, artificial intelligence

## Abstract

CT angiography has become, in recent years, a main evaluating modality for patients with coronary artery disease (CAD). Recent advancements in the field have allowed us to identity not only the presence of obstructive disease but also the characteristics of identified lesions. High-risk coronary atherosclerotic plaques are identified in CT angiographies via a number of specific characteristics and may provide prognostic and therapeutic implications, aiming to prevent future ischemic events via optimizing medical treatment or providing coronary interventions. In light of new evidence evaluating the safety and efficacy of intervening in high-risk plaques, even in non-flow-limiting disease, we aim to provide a comprehensive review of the diagnostic algorithms and implications of plaque vulnerability in CT angiography, identify any differences with invasive imaging, analyze prognostic factors and potential future therapeutic options in such patients, as well as discuss new frontiers, including intervening in non-flow-limiting stenoses and the role of CT angiography in patient stratification.

## 1. Introduction

Coronary artery disease (CAD) is the leading cause of death worldwide. This chronic process, related to atherosclerosis, is responsible for mortality as well as adverse events, including myocardial infarction (MI) and stroke [1]. Identifying CAD early is essential in patients’ management, as it could prevent the onset of ischemic events. The gold standard method for identifying CAD is invasive coronary angiography, which allows immediate assessment of the coronary tree anatomy, physiological assessment of identified stenoses, and intervention with stent or balloon angioplasty. Advancements in the field of computed tomography (CT) have introduced the field of cardiac CT angiography (CCTA), which allows a non-invasive assessment of CAD presence, especially in patients with chronic coronary syndromes (CCSs) [2]. CCTA is thus, in recent years, suggested as a first-line diagnostic test for ruling out obstructive CAD [3,4]. In support of the technique, the EVINCI study showed that, among different imaging techniques aiming to assess obstructive CAD, CCTA was the most accurate modality [5]. Furthermore, a CCTA-first strategy, as shown in the SCOT-HEART study, led to a reduction in MI and CAD-associated mortality at 5 years follow-up, without an increase in invasive testing [6]. These findings, however, did not translate into a significant clinical benefit, as reported in the PROMISE trial [7].

Atherosclerosis is an active inflammatory process with several distinct characteristics derived from intravascular imaging and histology studies. Identifying plaque parameters that categorize the plaque as “at-risk” for future events, with acceptable cost-effectiveness and, ideally, in a non-invasive manner, could alter preventive medicine practice and guide physicians into more intensified treatment. Advancements in CCTA image quality and spatial resolution have allowed researchers to identify such features and correlate them with both histology and intravascular imaging. Thus, the aim of this narrative review, through extensive research in three large medical literature databases, is to describe CCTA-identified high-risk plaque characteristics, analyze their prognostic impact, introduce advancements in the CT technology field that allow a quantitative approach to such measurements, and discuss potential therapeutic implication on patients with plaque vulnerability.

## 2. Vulnerable Plaque Pathophysiology

### 2.1. Atherosclerosis Progression

Atherosclerosis and consequently CAD is a chronic inflammatory process that initiates possibly from childhood and, over a number of years as precipitating risk factors increase, it ultimately leads to clinically recognized CAD and adverse outcomes, including acute coronary syndromes (ACSs). Its pathophysiology has been extensively described; however, a short overview is going to be provided, in order to set the foundations for vulnerable plaque formation [8,9]. The initial step of atherosclerosis requires endothelial dysfunction that will allow LDL accumulation into the intima layer [10], in which, along with factors such as shear stress, LDL itself has a key role [11]. However, specific LDL scavenger receptors may facilitate the transmigration process, with LDL and oxidized LDL (oxLDL) molecules being uptaken and transcytosed by endothelial cells through these receptors which then transmigrate to the arterial intima layer [12]. LDL and oxLDL intima accumulation leads to increased local inflammation. Specifically, macrophages recruited by endothelial activation [13] at the site of the atheroma internalize oxLDL via several receptors, resulting in the transformation of macrophages into foam cells, due to intracellular lipid accumulation. This process, as well as oxLDL itself, which is a well-recognized proinflammatory molecule [14], increases the production of local chemo- and cytokines, further creating an inflammatory environment. Further foam cell and LDL accumulation enhances the inflammatory response, via the nf-κB factor, and further facilitates endothelial activation, monocyte transmigration, and foam cell and cholesterol crystal formation, thus inducing the formation of the lipid core and the progression of atherosclerosis [15]. 

Several studies have identified the distinct characteristics that comprise the atherosclerotic lesion, including fibrous cap, lipid-rich pool, and necrotic lipid core and calcification. The fibrous cap is the developed fibrous tissue that covers the lipid core, in order to protect and support the increasing lipid pool and avoid prothrombotic phenomena. The fibrous cap is an important characteristic of atherosclerotic plaques, as a thin cap is a feature of vulnerability. In short, the physiologically present vascular smooth muscle cells (VSMCs), in the event of injury and in response to foam-cell secreted molecules [16], induce a healing process in the intima that results in an increase in extracellular matrix components and the creation of a fibrous cap, preventing (under normal circumstances) plaque rupture [17]. The lipid core is the main feature of atherosclerotic plaques, consisting of a lipid-rich region due to the accumulation of lipids. The differentiation of the lipid core into a necrotic lipid core mainly regards the absence of elastic and collagen fibers in the necrotic core [18]. Additionally contributing to the necrotic lipid is the infiltration of macrophages and subsequent macrophage apoptosis [19]. Finally, calcification has an incremental role in atherogenesis progression and stability. Atheroma progression and inflammation lead to pro-osteogenesis signaling, resulting in osteoblastic differentiation of VSMCs and vascular interstitial cells, and thus to the deposition of calcium in the atherosclerotic lesion [20,21]. Particularly important is the extent of calcification. In particular, macrocalcification (>5 mm) is more frequent in stable lesions found in deeper layers of the plaque that are surrounded by thick fibrous tissue, not close to the lumen, and not associated with disrupting plaque mechanics. Contrarily, microcalcifications and spotty calcification, which are most commonly identified near the lumen, are associated with plaque instability and vulnerability [22,23].

### 2.2. Plaque Vulnerability

The presence of vulnerable plaques was described more than 30 years ago, when Muller et al., whilst examining the impact of stressors on coronary events, found that there exists a distinct atherosclerotic plaque phenotype that is more prone to rupture and subsequent thrombosis and, thus, ACS [24]. Plaque vulnerability, also defined as high-risk plaque, was extensively researched, aiming to determine pathophysiological relations, prognosis, and uniform terminology [25]. Early studies tried to classify the distinct features of these lesions. They, thus, identified the so-called thin cab fibroatheroma (TCFA) feature by histology in several sudden cardiac death series, which was described as a distinctive lesion of the thin fibrous cap and large lipid core, associated with plaque rupture and thrombosis at these particular sites [26,27,28,29]. The pathophysiological mechanism of TCFA development has been of much discussion. It is estimated that inflammation in the plaque, and specifically T-cell induced interferon-γ production, greatly inhibits collagen production and deposition by VSMCs, while, provoked by the inflammatory environment, macrophages increase the production of matrix metalloproteinase (MMP), which degrades plaque collagen. Furthermore, as a result of altered expression of the telomeric repeat binding factor 2 and DNA damage, there is an increased VSMC senescence and apoptosis reported in these lesions, further limiting fibrotic tissue production capability [30]. These effects result in decreased protection of the plaque from rupture. Considering the ineffective plaque protection and increased local thrombogenicity, resulting from the macrophage-produced tissue factor and the increased production of fibrin and plasminogen activator inhibitor-1, due to the systemic effects of inflammation, TCFA features create an optimal environment for plaque rupture, thrombosis, and ischemic events [31].

Besides the distinctive plaque characteristics, flow characteristics also have a role in vulnerable plaque progression and future thrombotic event development. It is obvious that local hemodynamics influence plaque stability and therefore abnormal stress can turn into an increased risk of plaque rupture, as well as plaque progression. High shear stress is associated with VSMC apoptosis [32] and related TCFA parameters; thus, its pathophysiological role in TCFA development could explain adverse outcomes. Subsequent studies showed that, compared to intermediate shear stress, low stress enhances plaque and necrotic core progression, leading to more stenotic physiology, while high shear stress results in greater necrotic core and regression of fibrous and fibrofatty tissue, thus being related to a more vulnerable phenotype [33]. Abnormal (either high or low) shear stress was also recently found to be correlated with plaque vulnerability, as low shear stress was related to increased plaque burden and necrotic core, while increased shear stress with reduced fibrofatty and fibrotic plaque area [34]. Whether low and high shear stress are related to the same extent to plaque vulnerability formation has been questioned, as other studies suggest that high shear stress results in greater negative plaque remodeling compared to low (78 vs. 40%, *p* < 0.01) [35]. Thus, the role of shear stress in atherosclerotic plaques is essential, both for their development and subsequent clinical events.

## 3. CCTA Characteristics to Identify Vulnerable Plaques

CCTA is an essential tool in the non-invasive evaluation of angina symptoms and CCS, as it allows for the identification and characterization of stenotic lesions, thus estimating the individual patient risk and the need for more advanced non-invasive or invasive ischemia testing. Especially in the era of CT-Fractional Flow Reserve (CT-FFR) calculation during the CCTA, the final CCTA report can include important imaging and physiological information that could guide revascularization decision-making. CT-FFR has been shown to have a good correlation with invasive FFR, with both being able to discriminate obstructive from non-obstructive disease with similar efficacy [36]. Large randomized trials with both off-site and on-site measurements have showcased that measuring this parameter in stable chest pain patients and its addition to decision-making may improve referral to invasive angiography and lower costs [37,38,39]. Along with allowing for the identification of obstructive disease and associated ischemia, CCTA allows for the complete evaluation of the coronary arteries/plaques and, therefore, of specific plaque characteristics that categorize the plaque as “high-risk” for future events. Such characteristics include low attenuation, spotty or micro-calcifications, positive remodeling, and the napkin ring sign. Below, the significance and role of such characteristics in patient risk will be described.

CCTA characteristics that are considered high-risk include low attenuation plaque (LAP), positive remodeling, spotty calcifications, and the napkin ring sign (NRS). Of these, the only operator-dependent feature is NRS. According to a recently published expert consensus, the use of different cut-offs of Hounsfield units (HU) for the identification of LAP has been proposed. Specifically, a HU > 350 indicates dense calcium, 131 < HU < 150 indicates a fibrous plaque, 31 < HU < 130 indicates a fibro–fatty plaque, and −30 < HU < 31 indicates a necrotic core [40]. LAP is, therefore, indicated by the last category of HU < 30. Despite most researchers including this definition in their respective studies, other investigators indicate that a HU < 60 may better identify LAP [41]. However, the large trials, such as SCOT-HEART, included HU < 30 in their inclusion criteria [42], while the CAD-Reporting and Data System 2.0 (CAD-RADS 2.0) also defined LAP as HU < 30 in their reporting algorithm [43]. Positive remodeling is a relatively early compensatory atherosclerosis mechanism involving changes to the vessel wall and lumen enlargement in order to delay lumen obstruction. However, it is related to an extended lipid necrotic core and thus could serve as a marker of plaque instability [44,45,46]. Positive remodeling can be identified in CCTA as the presence of outward plaque growth, with the presence of atherosclerosis and minimal lumen loss, or as the ratio of outer vessel diameter at the site of plaque divided by the average outer diameter of the proximal and distal vessel being greater than 1.1 [43]. Spotty calcification or microcalcification, i.e., the presence of spotty calcified areas in atherosclerotic plaques, is also a high-risk feature that has been associated with TCFA and increased lipid index, while the number of calcifications can be associated with decreased fibrous cap thickness [23,47]. In CCTA, spotty calcifications are also visualized and defined as punctate calcium within a plaque (or calcium < 3 mm) [43]. Finally, NRS reflects the necrotic lipid core of an atherosclerotic plaque, a well-identified feature of vulnerable plaques, which is surrounded by a hyperattenuated ring-like area. NRS is thus a surrogate for TCFA and plaque vulnerability, which is recognized in CCTA in a noncalcified plaque cross-sectional image as a central area of low attenuation plaque in contact with the lumen and a ring-like peripheral hyperattenuation rim surrounding this area [43]. It is important to mention that NRS is the only qualitative finding in the assessment of high-risk plaques by CCTA. In the CAD-RADS 2.0 score, the presence of two or more high-risk features indicates a special marking in the score result [43].

In order for these markers to be used in research as risk factors for future events, the link between them and plaque vulnerability had to be confirmed. The first data regarding an association of CCTA parameters with plaque characteristics were reported in the early 2000s, with investigators documenting the accuracy of CT, in comparison with intravascular ultrasound (IVUS), in discriminating different plaque compositions in both stable and acute ischemia [48,49,50,51]. Choi et al., also in a comparative study of CT and IVUS, showed that CT attenuation was negatively correlated with necrotic core presence and positively with fibrous tissue, while the mean CT density values for plaques with greater or less than 10% necrotic core were 93.1 and 41.3 HU, respectively (*p* < 0.001) [52]. The first validation study came from the ATLANTA study by Voros et al. [53], which prospectively enrolled 60 patients and compared plaque features derived from CCTA and IVUS. Plaque geometry and composition were significantly correlated between the two modalities, while CCTA underestimated minimal lumen diameter and overestimated diameter stenosis. The compositional analysis of plaque features showed that high-attenuation non-calcified plaques correlated with fibrous tissue in IVUS, while low-attenuated plaques with the presence of necrotic core and fibrofatty tissue. These findings were further validated in ex vivo histology studies, showing a good discriminatory ability of CCTA and HU in identifying plaque vulnerability [54,55]. It is of interest that low attenuation plaques have been of increased prevalence in ST-elevation MI (STEMI) patients, compared to Non-STEMI (NSTEMI) patients, and have been associated with higher inflammatory marker levels, consistent with the plaque vulnerability pathophysiology [56]. Along with IVUS, CCTA and plaque attenuation have been compared with optical coherence tomography (OCT), showing good discriminatory ability for identifying plaque characteristics [57,58]. Finally, more recent studies have shown that increased high-risk intravascular imaging characteristics are independently associated with low attenuation, while low attenuation burden significantly increased with a similar increase in the number of intracoronary imaging high-risk features [59].

Moving on from hypoattenuation, NRS was also recognized relatively early as a marker of plaque vulnerability and a TCFA surrogate. Kashiwagi et al., assessing 105 CAD patients with CCTA and OCT, along with finding lower attenuation in TCFA plaques, also reported increased positive remodeling and the presence of a ring-like enhancement, with this being significantly correlated to TCFA in regression analyses [60]. Maurovich-Horvat et al. [61] also evaluated the association of NRS with atherosclerotic lesions in an ex vivo histology study of 21 coronary arteries which were also co-examined with CCTA. In contrast with non-calcified and mixed plaque categorization, NRS had an excellent specificity of 98.9% in diagnosing advanced atherosclerotic lesions, as well as a high positive predictive value. Similar histology studies have further established the association of NRS with a necrotic core and greater non-core plaque area, also indicating advanced, “at-risk” atherosclerotic lesions [62]. Moreover, IVUS studies have established the link between spotty calcification and high-risk features, with those plaques with the largest number of calcifications having the highest rate of TCFA presence [63]. Others have also stated the independent relationship of plaque vulnerability with spotty calcification [64]. Finally, plaque remodeling, as defined by a remodeling index greater than 1.1, has been described to associate well with high-risk plaques, compared to IVUS [65], with Kroner et al. [66] comparing IVUS and CCTA parameters, further associating positive remodeling derived from CCTA measurements with the presence of TCFA and a necrotic core. 

As mentioned, given the correlation of these characteristics with both vulnerable plaques and, as will be described below, with patient outcomes, the updated version of the CAD-RADS reporting system for CCTA mentions that identifying such factors in CCTA should be included in the report as a “modifier” (HRP indication), after the CAD-RADS score [43]. Further establishing these criteria, the Society of Cardiovascular Computed Tomography and the North American Society of Cardiovascular Imaging (SCCT/NASCI) published, in 2020, an expert consensus on the assessment of atherosclerotic plaque characteristics in CT, which highlights the association of the aforementioned analyzed characteristics with plaque vulnerability and outcomes, discusses technical considerations for optimal image quality and factors identification, suggests reporting these findings in a uniform way, and analyses potential implications for vulnerable plaque treatment [40].

Finally, it should be noted that the assessment of plaque vulnerability has certain limitations. These include CT spatial resolution, image quality in order to optimally identify differences in density and limit noise or artifacts, interobserver and interscan variability, and qualitative characteristics, such as NRS and plaque density overlap among distinct plaque characteristics [40]. Advancements in CT scanner technology and the use of artificial intelligence (AI) and machine learning (ML) could largely limit the limitations and the operator-dependent pitfalls of atherosclerosis assessment with CT.

## 4. Vulnerable Plaques Prognosis

It is well known that plaque vulnerability is a predictor of future events, as it is more prone to rupture and thrombosis. This has been identified early by histology studies, which found more high-risk characteristics in patients with ACS [26,27,28]. Intracoronary imaging studies have also associated high-risk plaque characteristics, in the absence of obstructive disease, with cardiovascular outcomes [67,68]. Establishing the relationship between CCTA plaque characteristics and vulnerability, several investigators aimed to evaluate whether those factors can also predict future events (Table 1).

Motoyama et al. [69] were among the first groups to assess whether high-risk CT characteristics can be linked to the development of ACS. As at the time LAP and positive remodeling were mostly evaluated, the investigators included these two factors in subsequent evaluations. They included 1059 patients, which were followed up for approximately 27 months. Out of 45 patients having both LAP and positive remodeling, 22.2% developed an ACS, in comparison to 3.7% and 0.5% in patients having only 1 or no feature, respectively; ACS, thus, was significantly predicted by the presence of remodeling and/or LAP with an HR of 22.8 (95% CI: 6.9–75.2).

The same group also evaluated these characteristics in longer-term follow-up, specifically, a mean time of 3.9 years [70]. They reported the occurrence of ACS in 16.3% of patients with high-risk characteristics and 1.4% of patients without. The highest rate of events was observed in patients with significant stenoses and high-risk plaques (19%), seconded by non-significant stenoses with high-risk plaque (15%), with LAP and positive remodeling being independent predictors of events. In a similar follow-up period (mean 3.3 years), Yamamoto et al. [71] also reported that despite non-calcified plaques being not solely predictive of events, non-calcification with the presence of low-attenuation and positive remodeling showed an adjusted HR for ACS events of 11.2 (95% CI: 3.71–36.7)

Nakanishi et al. [72] enrolled hypertensive patients with non-obstructive disease, evaluated them with CT, and tried to assess whether imaging parameters can predict ACS during a three-year follow-up period. In a total cohort of 134 patients, ACS occurred in 10 patients, with LAP being a significant predictor of events (*p* < 0.001). Regarding NRS, Otsuka et al. [73] evaluated its association with MACE. Most patients with NRS also had LAP or positive remodeling. During a mean 2.3-year follow-up, 2.6% of patients of the total cohort developed an ACS, with LAP, positive remodeling, and NRS being independent predictors of future events, while, in a Kaplan–Meier analysis, NRS plaques, in general, showed a significantly higher risk of ACS compared to non-NRS.

Hou et al. [74], investigated this correlation in a larger manner by following up 5007 patients with suspected CAD undergoing CCTA for a mean time of 3 years. At the time of the follow-up, both coronary artery calcium and plaque phenotype were related to major adverse cardiovascular events (MACEs), with the addition of those two factors to clinical risk within an area under the receiver-operating curve of 0.93 (*p* < 0.001).

The PROMISE study further evaluated the role of CCTA plaque parameters and their association with MACE [75]. The study enrolled 4415 stable, symptomatic CAD patients, with 15.3% of them having high-risk features. After a median of 25 months follow-up, vulnerability factors presence was associated with a higher MACE rate (6.4% vs. 2.4%; HR: 2.73; 95% CI: 1.89–3.93), with consistent results even after adjustment for atherosclerotic cardiovascular risk score and presence of significant stenosis. These features were even more predictive of MACE in women and younger individuals. In an effort to develop a risk prediction model in non-obstructive CAD from these results, Taron et al. [76] showed that composite events of unstable angina, non-fatal MI and mortality were independently predicted by atherosclerotic cardiovascular risk score (HR: 1.03), two or more high-risk plaque features (HR: 2.40) and stenosis severity 30–69% (HR: 1.91). In all assessed models and combinations, the addition of CCTA-identified plaque vulnerability significantly improved model fit, thus concluding that the addition of plaque composition in risk models may further discriminate the MACE risk of low–moderate risk asymptomatic CCS patients. 

Similarly, the ICONIC study examined the relation of atherosclerotic features with ACS. It included 234 ACS and non-CAD control patients with a CCTA who were followed up for a mean period of 3.4 years [77]. A total of 52% of patients had identifiable vulnerable plaques. Diameter severity, fibrofatty, necrotic core volume and high-risk features all increased the risk of developing ACS. Specifically, high-risk plaque presence was associated with an HR of 1.59 for ACS development (95% CI: 1.22–2.08), with LAP (HR: 1.38; 95% CI: 1.05–1.81) and spotty calcification (HR: 1.54; 95% CI: 1.17–2.04) also being significant predictors. Plaque remodeling showed a non-significant trend towards increased MACE (*p* = 0.085).

Another large CCTA study, the SCOT-HEART study, in which 34% of included individuals with stable chest pain had at least one vulnerable plaque feature in CCTA, also analyzed their correlation with MACE. Williams et al. [78] reported in this posthoc analysis that patients with vulnerable plaques had a 3-fold increased risk of CAD-associated mortality or nonfatal MI (HR: 3.01; 95% CI: 1.61–5.63), in contrast to a 2-fold increase in those with obstructive CAD (HR: 1.99; 95% CI: 1.05–3.79). The presence of both high-risk features and obstructive disease was associated with an HR of 11.50 for the aforementioned outcomes (95% CI: 3.39–39.04). However, it should be noted that these increases in mortality, despite being significant, were not found to be independent of coronary artery calcium score. Further investigating this relationship, the same groups reported the association of LAP with MACE [42]. LAP was not correlated with atherosclerotic cardiovascular disease risk score but was correlated with coronary artery calcium score and stenosis severity. LAP was found to be significantly higher in patients who experience MACEs (7.5% versus 4.1%; *p* < 0.001), and was the most significant independent predictor of MI (HR: 1.60; 95% CI: 1.10–2.34). This risk was further increased when the LAP burden exceeded 4% (HR: 4.65; 95% CI: 2.06–10.5). Finally, the investigators have observed that the predictive value of LAP is irrespective of gender [79].

Long-term follow-up associations have also become available in recent evaluations. Feucthner et al. [80] report outcomes on a mean 7.8 year follow-up. Regarding plaque vulnerability, CT density was significantly lower (35.2 HU ± 32 vs. 108.8 HU ± 53) in plaques in the MACE arm, while both NRS (63.4% vs. 28%) and LAP < 30, <60, and <90 HU (46.3–78% vs. 2.4–7%) were significantly more prevalent in those that developed MACE. Results from a mean 10.55-year follow-up were also provided by Senoner et al. [81], reporting that LAP, as defined by HU < 60, and NRS predicted MACE but not all-cause-mortality, after risk factor adjustment (HR: 4.00, 95% CI: 1.52–10.52 and HR: 4.11, 95% CI: 1.77–9.52, respectively). However, in contrast to previous results, spotty calcification and remodeling index did not significantly predict adverse events.

As previously mentioned, plaques exposed to increased wall shear stress and high-pressure gradients are most likely to develop high-risk characteristics, while including wall shear stress to stenosis severity improved discrimination of high-risk atherosclerotic lesions [82,83]. Aiming to evaluate the significance of adverse plaque features in relationship to coronary hemodynamic parameters, Yang et al. [84] performed a study including CCTA-derived hemodynamic and high-risk plaque factors and assessed their predictive value for MACE. The group found that, regardless of impaired or normal hemodynamics in the tested lesions, the presence of plaque vulnerability independently predicts MACE.

**Table 1:** Key studies on the prognostic effect of high-risk CCTA plaque characteristics.

Study	Year	Study Type	Setting	Number of Participants	Plaque Characteristic Assessed	Follow Up	ACS or MACE Prediction Rates	Other Outcomes
Motoyama et al. [69]	2009	Observational	Suspected CAD	892 patients	LAP, PR	27 ± 10 months	ACS rate was predicted by: Both features: 22.6% (10) One feature: 3.7% (1) None feature: 0.5% (4)	HRP independent predictor of ACS (HR: 22.8, 95% CI: 6.9–75.2)
Motoyama et al. [70]	2015	Observational	Suspected CAD	3158 patients	LAP, PR	3.9 ± 2.4 years	ACS rate: 16.3% (48) versus 1.4% (40)	Plaque progression independently predicted ACS, regardless of HRP(+) (27%) or HRP(-) (10%) compared to HRP(-)/PP(-) (0.3%)
Yamamoto et al. [71]	2013	Observational	Suspected CAD	453 patients	LAP, PR	3.3 ± 1.2 years	Adjusted HR for mortality, non-fatal MI and unstable angina: 11.2 (95% CI: 3.71–36.7; *p* < 0.0001)	-
Nakanishi et al. [72]	2012	Observational	Suspected CAD and hypertension	77 patients	LAP, PR	39 ± 10 months	The number of LAP (*p* < 0.001) was an independent predictor of ACS in multivariate analysis	Total LAP: 1.6 0.6 (ACS) vs. 0.05 0.23 (non-ACS) (*p* < 0.001) Total PR: 1.0 1.0 (ACS) vs. 0.1 0.3 (non-ACS) (*p* < 0.001)
Otsuka et al. [73]	2013	Observational	Suspected CAD	895 patients	LAP, PR, NRS	2.3 ± 0.8 years	2.6% developed ACS PR (*p* < 0.001), LAP (*p* = 0.007), and NRS (*p* < 0.0001) were independent predictors of future ACS	Events developed in 41% of NRS plaques
Ferencik et al. [75]	2018	RCT, secondary analysis	Stable, symptomatic CAD	4415 patients	LAP, PR, NRS	25 months	MACE rate: 3% HRP was associated with a higher MACE rate (6.4% vs. 2.4%; HR: 2.73; 95% CI: 1.89–3.93)	HRP increased MACE risk among patients with nonobstructive coronary artery disease compared to non-HRP (HR: 4.31 vs. 2.64; 95% CI: 2.25–8.26 vs. 1.49–4.69) HRP was a stronger predictor of MACE in women and younger patients
Taron et al. [76]	2021	Observational	Stable CAD	2890 patients	LAP, PR, NRS	26 months	Presence of two or more HRP features predicted MACE (HR: 2.40; *p* = 0.008)	Patients with ASCVD ≥ 7.5%, any HRP, and mild/moderate stenosis had significantly higher events than those not meeting these criteria (3.0% vs. 6.2%; *p* = 0.007)
Chang et al. [77]	2019	Observational	ACS and stable CAD	468 patients	PR, LAP, SC	3.4 ± 2.1 years	Future ACS events were predicted by: HRP presence: HR: 1.59; 95% CI: 1.22 to 2.08, LAP presence: HR: 1.38; 95% CI: 1.05–1.81 SC presence: HR: 1.54; 95% CI: 1.17–2.04	HRP was observed in 31.01% of future culprit lesion (LAP: 24.03%, PR: 76.74%, and SC: 17.83%)
Williams et al. [78]	2019	RCT, post-hoc analysis	Stable CAD	1769 patients	PR, LAP, SC, NRS	4.7 years (IQR 4.0–5.7)	MACE events: HRP 4.1% vs. non-HRP 1.4%; *p* < 0.001 In HRP, obstructive CAD: HRP 4.9% vs. non-HRP 2.4%; *p* = 0.036	Patients with HRP and obstructive disease had the highest event rate (HR: 11.50; 95% CI: 3.39–39.04; *p* < 0.001)
Feuchtner et al. [80]	2017	Observational	Suspected CAD	1469 patients	PR, LAP, SC, NRS	7.8 years	MACE group showed: Decreased LAP (35.2 HU ± 32 vs. 108.8 HU ± 53; *p* < 0.001) Increased NRS presence (63.4% vs. 28%; *p* < 0.001) LAP < 30, <60, and <90 HU presence (46.3–78% vs. 2.4–7%; *p* < 0.001).	After adjustment, LAP < 60 and NRS were independent predictors of MACE
Senoner et al. [81]	2020	Observational	Suspected CAD	1430 patients	PR, LAP, SC, NRS	10.55 ± 1.98 years	MACE, but not mortality, were predicted by: LAP < 60 HU (HR: 4.00, 95% CI: 1.52–10.52, *p* = 0.005) NRS (HR: 4.11, 95% CI: 1.77–9.52, *p* = 0.001) Non-significance of SC and PR	HRP features, when added to CAD-RADS and calcium score for MACE prediction, perform superior to solely calcium score (c = 0.816 vs. 0.716, *p* < 0.001)
Yang et al. [84]	2022	Observational	Stable CAD	335 patients	LAP, PR	2.9 years	MACE were predicted by HRP (HR: 2.70; 95% CI: 1.10–6.50; *p* = 0.02)	HRP predicted MACE irrespective of flow or pressure gradient parameters

Abbreviations: CAD: Coronary Artery Disease; ACS: Acute Coronary Syndrome; MACE: Major Adverse Cardiovascular Events; HRP: High Risk Plaque; LAP: Low Attenuation Plaque; PR: Positive Remodelling; NRS: Napkin Ring Sign; SC: Spotty Calcification; CAD-RADS: Coronary Artery Disease-Reporting and Data System; HU: Hounsfield Units; MI; Myocardial Infarction; PP: Positive Remodelling; HR; Hazard Ration; CI: Confidence Interval; ASCVD: Atherosclerotic Cardiovascular Disease.

Finally, evidence for the predictive value of plaque characteristics in MACE in acute chest pain became recently available through the RAPID-CCTA study [85]. Meah et al. reported that, in the patients that had a future event, non-calcified plaque and LAP were significantly increased, with total, non-calcified plaque and LAP burden being the strongest predictors of MACE independently of the presence of obstructive CAD and GRACE score. Interestingly, a LAP above the median was associated with an HR of 7.80 (95% CI: 2.33–26.0) for 1-year mortality or non-fatal MI, outperforming both obstructive CAD and GRACE scores.

Synthesizing more recent trials with older data, Gallone et al. [86] provided an updated meta-analysis on the value of high-risk characteristics in predicting MACEs of non-obstructive CAD. They included a total of 30 studies, with 9 being prospective, and 30,369 patients. A total of 21 studies included data from CCTA studies, while others included data from intravascular imaging trials. The study showed good predictive ability of CCTA-derived features for prediction of MACE, as well as for the combination of high-risk plaque features, which were also comparable with intravascular imaging results. Thus, available evidence showcases the importance of CCTA and plaque characterization for predicting adverse events and justifying the inclusion of such features in risk scores, such as CAD-RADS 2.0.

## 5. High-Risk Features Quantification: The Use of AI, ML and Radiomics

Given the operator-dependent and time-consuming assessment of plaque characteristics, it is evident that novel technological advancements that integrate identifying and reporting such features with automatic AI and ML algorithms aiding this recognition would lead to quantitative measurements with large implications for clinical practice. It is notable that, in comparison with AI, expert readers of CCTA had significant discordance and variability [87], which increases with the use of semi-automatic software [88]. As implementing AI, ML and radiomics into everyday clinical practice could largely increase diagnostic ability and limit CCTA analysis time, several novel investigations have tested the feasibility of these technologies in predictive models and high-risk characteristics recognition (Table 2).

### 5.1. AI and ML

Masuda et al. [89] used ML to determine whether an ML histogram analysis of CCTA characteristics has a better diagnostic yield for plaque composition characterization than conventional methods. Including 78 CCTA patients undergoing CCTA and IVUS, the ML algorithm showed a significantly higher area under the curve (AUC) than the conventional method (0.92; 95% CI: 0.86–0.92 versus 0.83; 95% CI: 0.75–0.92; *p* = 0.001), highlighting the efficacy of ML in discriminating fibrous and fatty/fibrofatty plaques. Similarly to these results, the CLARIFY study by Choi et al. [90] showed that AI detected more high-risk plaque features in CCTA (21.1%) compared to consensus expert readers (13.4%), with, however, a good agreement between modalities (82%). Importantly, the investigators note the rapid assessment of plaque characteristics by AI, with a mean time of 10 min being reported. 

Following this, Lin et al. [91] used the SCOT-HEART cohort to develop and validate a deep-learning system for identifying plaque characteristics and stenosis grades. A novel neural network was trained extensively (approximately 5000 lesions) and then was applied to a second test set, including external validation CCTA and IVUS-assessed cohorts. Overall, there was a good agreement between the deep-learning and expert imager measurement of both stenosis grade and plaque volume [intraclass correlation coefficient (ICC) 0.879 and 0.964, respectively], as well as a good correlation with IVUS-derived characteristics and measurements. Regarding prognostication, a derived-by-deep learning plaque volume greater than 238.5 mm^2^ was associated with MI events (HR: 5.36; 95% CI: 1.70–16.86), after adjustment. Importantly, there was a significant difference in the plaque analysis time by deep learning (mean 5.56 s) and expert imagers (mean 25.66 min). 

Further studies aimed to examine the identification of high-risk plaque features by AI and deep learning and ACS prediction. Koo et al. [92], in the EMERALD-II trial, investigated the diagnostic value of AI-enabled quantitative plaque analysis, which is also associated with hemodynamics in ACS patients with previous CCTA. The best identified features, which were included in the model, were plaque burden, total plaque volume and low-attenuation plaque volume, along with hemodynamic parameters. Subsequently, in the tested validation cohort, this model showed a higher prediction of future ACS plaques than conventional methods, with an AUC of 0.84 compared to 0.78 (*p* < 0.001), regardless of the time point of CCTA before the ACS event. 

Finally, Tzimas et al. [93], with the use of AI, developed nomographic quantitative plaque values based on age and gender. Evaluating 11,808 patients, they reported that the median total plaque volume was 223 mm^3^ (IQR: 29–614 mm^3^), which was significantly greater in males (360 mm^3^ versus 108 mm^3^; *p* < 0.0001) and increased in both genders with age. Total plaque increased with age in both male and female patients, while younger patients had a higher prevalence of noncalcified plaques. These data for the first time provide nomograms allowing better quantitative analysis of plaque volume, with potential extensions of this work in high-risk plaque characteristics quantification, with the use of AI being of outmost importance for the integration of fully automated quantitative algorithms into clinical practice.

### 5.2. Radiomics

Radiomics is a novel approach in cardiovascular and medical research, representing a method for quantitative analysis of medical imaging. More specifically, radiomics aims at enhancing the existing data available to clinicians by means of advanced, and sometimes non-intuitive, mathematical analysis [94]. Its applications in cardiovascular disease are emerging and, thus, investigators assessed whether it can be of use in high-risk plaque assessment.

Kolossvary et al. [95] investigated whether radiomics can improve the identification of the, relatively operator-dependent, NRS. The study reported that 20.6% (916/4440) of radiomic features were significantly different between NRS and non-NRS plaques, with approximately half of them having an AUC greater than 0.80. The highest AUC was shown by short-run low gray-level emphasis, long-run low gray-level emphasis and surface ratio of high attenuation voxels to total surface. 

Moreover, Lin et al. [96] assessed if a radiomic signature can be overall associated with high-risk plaque characteristics, finding that radiomics features could discriminate with high efficacy lesions with HRP, compared to conventional qualitative techniques (AUC 0.86 vs. 0.76; *p* = 0.004). Similar positive results were also identified in an ex vivo study, where radiomics-based ML assessment significantly outperformed visual coronary features assessment [97].

Finally, some studies further linked radiomic features with MACE and plaque progression. Specifically, Chen et al. [98] found that a radiomic signature was an independent predictor of rapid plaque progression, with an odds ratio of 2.35 (95% CI: 1.32–4.4), even after cofounder adjustment. Regarding MACE, the same group [99] showed that a 16-radiomic feature model associated with plaque vulnerability independently predicted MACE at 3 years of follow-up. Given these important findings, further evaluation of radiomic features could provide an enhanced path for identifying high-risk plaques and better predict outcomes than visual, qualitative feature assessment. Such questions will be more elaborately answered when more evidence from large trials becomes available in the forthcoming years.

**Table 2:** Key studies on the use of AI, ML and radiomics in the CCTA assessment of high-risk atherosclerotic plaques.

Study	Year	Study Type	Type of Modality Used	Comparator Group	Number of Participants	Plaque Characteristic Assessed	Outcomes
Artificial Intelligence and Machine Learning							
Masuda et al. [89]	2019	Observational	ML, CCTA	Median cut-off CT number	78	Fatty–Fibrofatty composition	ML-yielded results showed a significantly higher AUC compared to the conventional measurement (ML-AUC: 0.92; 95% CI: 0.86–0.92 vs. Conventional-AUC: 0.83; 95% CI: 0.75–0.92; *p* = 0.001).
Choi et al. [90]	2021	Observational	AI, CCTA	CCTA expert readers	232	CAD-RADS HRP Features	HRP features were recoginzed in 21.1% by AI vs. 13.4% by expert readers (82% agreement)
Lin et al. [91]	2022	Observational	ML, CCTA	CCTA expert readers	1196		Good agreement regarding plaque volume [ICC 0.964] and good correlation with IVUS-derived characteristics. ML-derived plaque volume > 238.5 mm^2^ associated with MI (HR: 5.36; 95% CI: 1.70–16.86) Significant less time for plaque analysis by ML (mean 5.56 s vs. 25.66 min).
Koo et al. [92]	2024	Observational	AI, CCTA	Reference CAD-RADS model	351	CAD-RADS and AI-derived feauters (relation with ACS prediction model)	Best predictive HRP features (included in the AI model): plaque burden, total plaque volume, low-attenuation plaque volume Validation: Higher prediction of future ACS-related plaques than conventional reference methods (AI-AUC: 0.84 VS Reference-AUC: 0.78; *p* < 0.001)
Tzimas et al. [93]	2024	Observational	AI, CCTA	N/A	11,808	Plaque volume (AI-identified normographic values)	Significantly higher plaque volume in males compared to feales (360 mm^3^ vs. 108 mm^3^; *p* < 0.0001). Total plaque volume increased with age in both genders.
Radiomics							
Kolossvary et al. [95]	2017	Observational	Radiomics, CCTA	Conventional NRS sings	60	NRS	No conventional sign significantly differentiated NRS from non-NRS 20.6% of radiomic features were signifcicantly different in NRS vs. non-NRS, with 50% of these having an AUC > 0.80
Lin et al. [96]	2022	Observational	Radiomics, CCTA	Conventional qualitative HRP measurement	60	Conventional HRP characteristics	Radiomic features identify HRP lesions with higher efficacy compared to conventional qualitative techniques (AUC 0.86 vs. 0.76; *p* = 0.004).
Kolossvary et al. [97]	2019	Observational (ex vivo)	Radiomics, CCTA	Histology assessment	7	Fatty and fibrous tissue composition; HRP characteristics	The radiomics model outperformed visual assessment (AUC = 0.73 vs. 0.65; *p* = 0.04) and histogram-based areas of low attenuation (AUC = 0.55 vs. 0.68; *p* = 0.01) in the assesment of advanced atherosclerosis
Chen et al. [98]	2023	Observational	Radiomics, CCTA	Conventional CCTA morpological assesment	214	Rapid plaque progression	Radiomics significantly outperformed morphological assesment
Chen et al. [99]	2023	Observational	Radiomics, CCTA	IVUS	255	HRP features and relation to prognosis	The radiomics model had moderate–good AUCs from training through calidation, internal and external sets (0.81, 0.75, 0.80 and 0.77). Radiomic signature equal or greater to 1.07 was significantly related to MACE at 3 years (HR: 2.01)

Abbreviations: AI: Artificial Intelligence; ML: Machine Learning; CCTA: Coronary Computed Tomography Angiography; CT: Computed Tomography; AUC: Area Under the Curve; HRP: High-Risk Plaque; ICC: Intraclass Correlation; ACS: Acute Coronary Syndrome; NRS: Napkin Ring Sign; CAD-RADS: Coronary Artery Disease—Reporting and Data System; IVUS: Intravascular Ultrasound; MACE: Major Adverse Cardiovascular Events; HR: Hazard Ratio; 95% CI: 95% Confidence Interval.

## 6. Management of Vulnerable Plaques: Potential Indications

### 6.1. The Effect of Statin Treatment

The identification of vulnerable plaques has been a long-standing evaluation method in order to identify risk scores and pharmacotherapy options toward either plaque stabilization or regression. Statins were the main investigated approach for multiple years regarding plaque feature alterations. Extensive research has shown that, in patients with CAD, high-intensity statin treatment prevented the progression of atherosclerosis, as shown by the percentage change in atheroma volume (PAV) and other IVUS-identified parameters [100]. Similar results were shown in patients receiving early statin treatment after an ACS, with significant changes in plaque volume associated with the LDL reductions [101], while no difference in plaque progression reversal has been identified with the use of different high-intensity statins [102]. Following this, it was identified that lipid-lowering not only results in a reduction in plaque progression but also in plaque regression. Specifically, the ASTEROID trial, assessing the relationship of rosuvastatin treatment with IVUS-related plaque regression surrogates, reported that statin treatment was associated with a significant reduction in all plaque regression markers, namely, percent atheroma volume, nominal atheroma volume and change in the normalized total atheroma volume [103].

CCTA has also largely been used to evaluate the impact of statins on plaque regression. Lee et al. [104], in the PARADIGM study, showed that statin use, compared to statin-naiveness, was associated with a lower progression of PAV per year (1.76 ± 2.40% vs. 2.04 ± 2.37%; *p* = 0.002) and increased plaque calcification. Furthermore, the incidence of new high-risk plaque features was reduced in the statin group (0.9% vs. 1.6% per year, *p* < 0.001). Thus, statin use was related to a 21% reduction in PAV progression and a 35% HRP development reduction above the median. However, those with increased baseline plaque and plaque vulnerability burden seem to not benefit from the treatment in recent analyses [105]. Similar results, compared to placebo, were shown by other groups as well [106]. 

### 6.2. The Use of PCSK9 Inhibitors

Given the association of plaque stabilization and regression with LDL levels, it was logical to assume that the greater, immediate LDL reduction provided by the PCSK9 inhibitor use could greatly influence atherosclerosis progression, as was initially hypothesized in ACS patients [107]. The first data on the positive effect of PCSK9 inhibition on atherosclerotic plaques were observed by Nichols et al. when evaluating evolocumab in the GLAGOV study [108]. After 76 weeks of treatment, among the 968 patients enrolled in the study, and, in comparison to placebo, evolocumab resulted in lower LDL levels as well as a significant decrease in PAV. In addition, normalized total atheroma volume (TAV) also significantly decreased in the evolocumab group, with a mean difference of −4.9 mm^3^ (95% CI, −7.3 to −2.5; *p* < 0.001). Thus, the investigators reported that evolocumab resulted in a greater plaque regression compared to placebo, showcasing its effect on plaque modification.

Following this, the HUGYENS study aimed to assess the effect of evolocumab on plaque characteristics in post-MI patients [109]. The investigators included patients with non-ST segment elevation MI (NSTEMI) who either received evolocumab or a placebo for 52 weeks on top of statin treatment. Plaque characteristics were assessed with OCT. At the time of the follow-up, those who received evolocumab showed a significant increase in minimum fibrous cap thickness (+42.7 vs. +21.5 μm; *p* = 0.015) and a decrease in maximum lipid arc (−57.5° vs. −31.4°; *p* = 0.04). Moreover, there was a greater regression of PAV in the PCSK9 inhibitor arm (−2.29% ± 0.47% vs. −0.61% ± 0.46%; *p* = 0.009), with no noted difference in calcium between groups.

The PACMAN-AMI [110] was a randomized study that investigated the effect of alirocumab in addition to statin therapy in atherosclerotic characteristics after MI. The study included 300 patients, with 148 receiving alirocumab and the rest placebo, less than 24 h after percutaneous coronary intervention (PCI). IVUS, OCT and near-infrared spectroscopy (NIRS) were used to assess plaque composition at baseline and at 52 weeks follow-up. In concordance with the aforementioned studies, the investigators reported a significant mean change in PAV (difference, −1.21% [95% CI, −1.78% to −0.65%], *p* < 0.001), LCBI_4mm_ (difference, −41.24 [95% CI, −70.71 to −11.77]; *p* = 0.006) and minimal fibrous cap thickness (difference, 29.65 μm [95% CI, 11.75–47.55]; *p* = 0.001). Regarding adverse events, no significant difference was identified.

Finally, the ARCHITECT study evaluated PCSK9 inhibitor-associated plaque modification on CCTA features of patients with familial hypercholesterolemia. Perez de Isla et al. [111] assessed 104 patients treated with alirocumab for 78 weeks and compared baseline and follow-up CCTA high-risk characteristics. At follow-up, coronary plaque burden significantly decreased from 34.6% at baseline to 30.4% (*p* < 0.001), while an increase in the proportion of calcified (+0.3%; *p* < 0.001) and mainly fibrous (+6.2%; *p* < 0.001) plaque, and a decrease in the percentage of fibro–fatty (–3.9%; *p* < 0.001) and necrotic plaque (–0.6%; *p* < 0.001), were also noted. These characteristics, identified by lipid plaque attenuation at CCTA, as described, showed the positive effect of PCSK9 inhibitors in vulnerable plaque regression and stabilization.

It is of interest that these reductions in coronary plaque adverse features are also associated with a survival benefit. Recently, a PACMAN-AMI substudy reported that a “triple-regression” phenotype of the atherosclerotic plaque, which was independently predicted by treatment with a PCSK9 inhibitor, resulted in a significant reduction in the composite of mortality, MI and ischemia-driven revascularization [112]. Given the positive effects of PCSK9 inhibition in plaque characteristics, LDL levels and MACEs, it is largely supported that they should be started early, especially after ACS, in order to maximize benefit and stabilize any prone-to-rupture lesion [113]. A similar indication could be patients with significant high-risk plaque presence, who, as they are at an increased rate of subsequent events, could potentially benefit from plaque regression and, ultimately, prevent ischemia. This suggestion should be properly investigated in well-powered trials in order to provide evidence regarding prophylactic extensive lipid lowering based on imaging criteria.

### 6.3. Preventive Interventional Plaque Modification

Despite the fact that pharmacotherapy has proven thus far to facilitate plaque stabilization and regression, the suboptimal pharmacotherapy in a major proportion of patients as well as the increased risk of events and mortality associated with high-risk plaques led to the hypothesis that intervening in non-flow limiting, non-significant coronary lesions with high-risk plaque characteristics identified at intravascular imaging would be of benefit as a preventive strategy. This hypothesis led to the design of early feasibility studies, testing the role of preventive PCI in vulnerable plaques, with many trials still ongoing, but initial results also being reported this year.

The first study to evaluate stenting in high-risk, non-significant coronary was the PROSPECT ABSORB trial by Stone et al. [114]. The investigators, after identifying non-culprit, high-risk, non-flow limiting stenoses by IVUS and NIRS in the three coronary vessels of MI patients, randomized the patients to either undergo PCI with a bioresorbable vascular scaffold (BVS) on top of medical therapy (93 patients) or only receive medical therapy (89 patients). At the time, optimal medical therapy did not include PCSK9 inhibitors. At 25 months follow-up, the minimum lumen area (MLA) in PCI-treated lesions was 6.9 ± 2.6 mm^2^ versus 3.0 ± 1.0 mm^2^ in the medical therapy arm (mean difference: 3.9 mm^2^; 95% CI: 3.3 to 4.5; *p* < 0.0001). Target lesion failure (TLF) at 24 months was similar between arms, as was lesion-related MACE, with a trend towards the benefit of PCI (odds ratio: 0.38; 95% CI: 0.11 to 1.28; *p* = 0.12). However, it has to be noted that the study was not powered to assess clinical events.

The first major results on this topic came recently from the first randomized trial, PREVENT. Park et al. [115] included patients with non-low limiting, high-risk lesions as defined by intravascular imaging features (MLA < 4.0 mm^2^ and/or plaque burden > 70% and/or large lipid-rich plaque and/or TCFA), with the requirement of at least one high-risk criterion. The primary endpoint of the study was a composite of cardiovascular death, target vessel MI, ischemia-driven target-vessel revascularization, or hospitalization for unstable or progressive angina at 2 years. A total of 1606 patients equally assigned to PCI and medical therapy or medical therapy alone were recruited. Similarly to the aforementioned trial, PCSK9 inhibitors were not included in the design of the study. At the time of the follow-up, the primary endpoint occurred in three (0.4%) patients in the preventive PCI and in 27 (3.4%) patients in the medical therapy arm (absolute difference: −3.0 percentage points; 95% CI −4.4 to −1.8; *p* = 0.0003). Preventive PCI performed consistently better than medical treatment alone in each component of the primary outcome. Finally, the adverse event rate did not differ between the two arms, highlighting the safety and efficacy of preventive PCI in high-risk plaques for limiting future events. However, it is of note that, after 2 years, the rate of increase of the primary outcome was similar between the two arms, possibly indicating that, after two years, previously unidentified high-risk plaques progressed, resulting in ischemic events. This observation, despite explaining the similar rate between the two cohorts, requires further evaluation. 

Finally, the “leave nothing behind” concept, i.e., the use of drug-coated balloons (DCBs) rather than stents, seems to be even more appealing in the setting of preventive PCI, as it would limit the need for post-procedural ischemic and bleeding events. Towards this direction, van Veelen et al. [116] published the first report of the DeBut-LRP trial, which aims to assess the safety and feasibility of paclitaxel DCB in non-flow limiting, non-culprit high-risk stenoses of patients with NSTEMI. A total of 20 patients were enrolled, and 14 received a DCB, as the other 6 had suboptimal anatomy. Three procedural complications occurred (one in non-culprit and two in culprit lesions). At follow-up imaging, the primary endpoint of the median maximum lipid core burden index (LCBI) in a 4 mm segment (maxLCBI_4mm_) was assessed and was significantly decreased from 397 (IQR 299 to 527) at baseline to 211 (IQR 106 to 349) at follow-up (*p* < 0.001; absolute change: −131 and relative change: −42%). The MLA also trended towards an increase but did not reach statistical significance. Five cardiovascular events were noted, with none being associated with DCBs. Unfortunately, given the small sample and the absence of a control arm, no conclusions can be drawn regarding the comparison of a DCB strategy with optimal pharmacotherapy. However, well-designed and powered ongoing and future trials will soon provide more insight into the intriguing field of preventive PCI for vulnerable plaques. 

## 7. Future Directions

**Figure 1:**
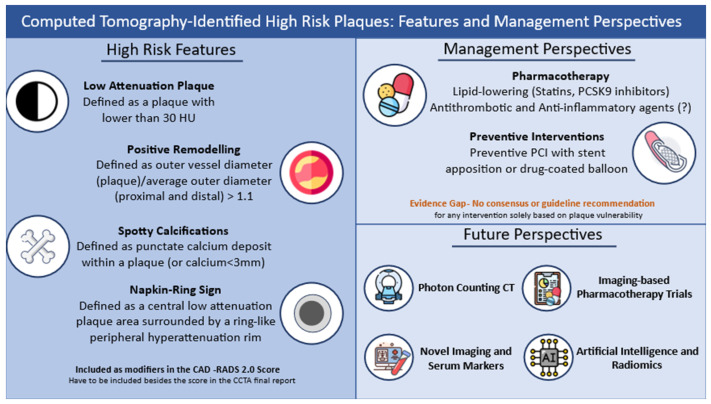
Computed tomography-identified high-risk plaques: features and management perspectives. Abbreviations: HU: Hounsfield Unit; CCTA; Coronary Computed Tomography Angiography; PCI: Percutaneous Coronary Intervention; CT: Computed Tomography.

Plaque vulnerability is an important precursor of acute ischemic events. As described, proper identification of markers indicating a high-risk plaque and, subsequently, future MACE and mortality is essential in order to prevent adverse events and improve patient outcomes (Figure 1). CCTA and the classification of such features come as an important diagnostic test, especially as it is recommended for low–moderate CAD risk patients as the first assessment of coronary obstructive disease [3,4]. Given the high usability of this examination and the described high rate of high-risk features identified during this examination, as shown by large studies, it is essential to implement reporting these features in CCTA reports and take them into consideration in clinical decision-making. However, some limitations of the aforementioned studies have to be mentioned. These include the observational design in most of them as, while the RCT studies were mostly posthoc analyses, the different populations and definitions of plaque characteristics, and the dependence on qualitative measurements, especially regarding NRS, are highly dependent on the center and its experience, as well as the lack of official consensus on trial outcomes and endpoints in such studies. Furthermore, the present CT technology has limited spatial resolution compared to the newer methods described below as well as compared to high-resolution invasive imaging of plaque vulnerability. Such limitations have to be taken into account in future studies, while the implementation of AI and radiomics could provide even more benefits and reproducibility in the analysis of plaque characteristics. Currently, no considerations for treatment in asymptomatic individuals with non-obstructive CAD and vulnerable plaques exist. On the other hand, recently, a consensus document of American cardiological societies was published analyzing the role of lipid-lowering based on CCTA-derived coronary artery calcium [117]. Similar guidance and recommendations should be available for high-risk features, given their prognostic effect on MACE. Further trials, assessing treatment strategies in patients with vulnerable plaques, including lipid-lowering, antithrombotics, anti-inflammatories and interventional techniques, should, therefore, be designed in order to guide clinical decision-making. 

Except from technological advancements in the field of AI and radiomics, improvement in CT technology could also provide a benefit in high-risk feature assessment. Photon counting CT (PCCT) is a new detector technology, introduced in 2021, that is based on semiconductor-composed CT detectors and converts photons directly and individually into electron-hole pairs, proportionally to the photon’s energy. This method allows for increased spatial resolution, thus allowing more precise information detection and reduced noise as well as radiation dose reduction [118]. Only a limited number of different systems are currently commercially available, with more being actively tested. Early CCTA studies with PCCT showed improved spatial resolution, which resulted in better identification of stented areas, small branches and the presence of calcification compared to conventional technology [119,120], as well as improvements in calcium volume and Agatston score [121]. Furthermore, studies in coronary lesion characteristics, in comparison to ordinary CT, have shown an increased diagnostic ability for both calcified and non-calcified plaques, as well as lipid-rich and fibrotic plaques [119,122,123]. However, it should be mentioned that the impact of this modality in clinical decision-making, and whether the increased image quality justifies the use of PCCT and its limitations, still remains largely unanswered, especially with regard to plaque-vulnerability identification. The rapid evolvement in this field and future comparative studies with both CT and intravascular imaging will provide more data regarding significant differences in plaque characterization, which could potentially lead to important changes in clinical practice.

The discussion of extensive, or even invasive, treatment of atherosclerotic plaques with high-risk features is still ongoing, with evidence of plaque stabilization with the use of extensive lipid-lowering therapies, among them PCSK9 inhibition. Further studies expanding our knowledge on the currently tested agents, as well as novel agents such as inclisiran, will provide further insight into the timing, benefit and indications of treating the plaque vulnerability phenotype. Importantly, on top of lipid-lowering agents, novel drugs such as sodium–glucose cotransporter 2 inhibitors (SGLT2is) and glucagon-like peptide 1 (GLP-1) analogs have shown positive plaque modification. Intravascular imaging studies have shown that SGLT2i treatment resulted in significantly increased fibrous cap thickness and decreased lipid arc degree and microphage grade [124], as well as a greater reduction in PAV [125]. Similar results have been shown in the preclinical setting for GLP-1 analogs [126]. More evidence and clinical studies’ results are awaited for both GLP-1 analogs, as well as colchicine, in the near future [127,128].

Finally, besides intracoronary plaque characteristics, novel CT markers, such as epicardial fat, can be associated with high-risk plaques and MACEs. Recent evidence suggests that increased epicardial adipose tissue is significantly associated with the presence of high-risk plaques [129], while its thickness has been correlated to lipid core burden [130]. Studies also suggest that epicardial adipose tissue is related to MACE [131,132]. Similarly, perivascular adipose tissue (PVAT) can serve as a potential marker of inflammation, which further promotes plaque vulnerability. Vascular inflammation and the inhibition of atherogenesis in favor of lipolysis leads to an increase in the lipocytes’ water content. This effect, along with edema, leads to the overall attenuation of PVAT and thus results in a marker of coronary inflammation [133]. Identifying novel imaging and serum markers of plaque vulnerability and associating them with existing vulnerable plaque features will enhance diagnostic performance and add more value to the overall risk assessment of patients. Further research with promising markers is, therefore, a key step towards establishing vulnerable plaque assessment and, potentially, treatment, based on non-invasive techniques. 

## 8. Conclusions

High-risk coronary atherosclerotic plaques and their distinctive CCTA features hold major significance for clinical practice. Their excellent correlation with histology and intracoronary imaging parameters, as well as their prognostic value, has established their reporting in standard CCTA reporting scores in order to inform physicians regarding individualized patient risk even in the absence of stenosis. Advancements in AI, radiomics and CT technology, as well as future trials in the preventive, imaging-guided treatment of plaque vulnerability, are promising research frontiers in the pursuit of further establishing these parameters in everyday practice and risk estimation.

## Data Availability

No new data were created, as this is a review article.
